# Sustained Activity of Metabotropic Glutamate Receptor: Homer, Arrestin, and Beyond

**DOI:** 10.1155/2017/5125624

**Published:** 2017-11-21

**Authors:** Geehoon Chung, Sang Jeong Kim

**Affiliations:** ^1^Department of Physiology, Seoul National University College of Medicine, Seoul, Republic of Korea; ^2^Department of Brain and Cognitive Sciences, Seoul National University College of Natural Sciences, Seoul, Republic of Korea; ^3^Department of Biomedical Sciences, Seoul National University College of Medicine, Seoul, Republic of Korea; ^4^Neuroscience Research Institute, Seoul National University College of Medicine, Seoul, Republic of Korea

## Abstract

When activated, metabotropic glutamate receptors (mGlus) exert long-lasting changes within the glutamatergic synapses. One mechanism is a tonic effect of downstream signal transduction pathways via sustained activation of mGlu itself. Like many other G protein-coupled receptors (GPCRs), mGlu can exist in a constitutively active state, which persists agonist independently. In this paper, we review the current knowledge of the mechanisms underlying the constitutive activity of group I mGlus. The issues concerning Homer1a mechanism in the constitutive activity of group I mGlus and recent findings regarding the significant role of *β*-arrestin in sustained GPCR activity are also discussed. We propose that once in a state of sustained activation, the mGlu persistently activates downstream signaling pathways, including various adaptor proteins and kinases, such as *β*-arrestin and mitogen-activated protein kinases. In turn, these effector molecules bind to or phosphorylate the mGlu C-terminal binding domains and consequently regulate the activation state of the mGlu.

## 1. Introduction

Efficient transmission of information in the nervous system is mediated by various neurotransmitters and neuromodulators. Glutamate, the most abundant neurotransmitter in the nervous system, acts as an excitatory signal in the synapses and plays a key role in the regulation of neuronal activity. In the synaptic loci, glutamate released from presynaptic vesicles binds to postsynaptic glutamate receptors, and synaptic activation of the postsynaptic ionotropic glutamate receptors, such as N-methyl-D-aspartate (NMDA) and *α*-amino-3-hydroxy-5-methyl-4-isoxazolepropionic acid (AMPA) receptors, directly contributes to the generation of action potentials in the postsynaptic neurons. Activation of the metabotropic glutamate receptor (mGlu), on the other hand, exerts indirect long-lasting influences throughout the neuron, primarily via the activation of heterotrimeric G proteins [1–3].

Based on intracellular signaling pathways, the eight subtypes of mGlus can be classified into three subgroups (I, II, and III). Among the eight mGlu subtypes, the most extensively studied mGlus are mGlu_1_ and mGlu_5_, which constitute group I mGlus [4, 5]. The activation of group I mGlus stimulates phospholipase C (PLC) *β*, resulting in activation of a diacylglycerol- (DAG-) mediated protein kinase C (PKC) pathway, and exerts calcium response by facilitating the opening of plasma membrane calcium channels and the release of inositol triphosphate- (IP_3_-) mediated calcium from the intracellular calcium stores [6]. The intracellular signaling cascades activated by group I mGlus play an essential role in the plasticity of neuronal excitability [6]. This is achieved by endocannabinoid-mediated suppression of presynaptic vesicle release probability, modulation of the receptor and channel availability in the postsynaptic neuronal membrane, and alteration in the transcription of genes related to various regulatory signaling molecules [5].

Akin to many other GPCRs [7–10], mGlus exist in a state of equilibrium between being active or inactive, regardless of agonist binding [11–13]. As such, mGlus can show sustained activity under certain circumstances. The persistence of mGlu activity after agonist washout as well as the constitutive mGlu activity independent of agonist binding has been reported in previous studies [13–15]. The sustained cellular effects of mGlu activation are mediated by downstream effectors, including G proteins or *β*-arrestins, and play a critical role in modulating neuronal plasticity [6, 16–18]. Further, previous studies have reported that the persistent effect of mGlu activation is involved in physiological function and pathological dysfunction of the nervous system [11, 14, 19].

In this review, we will focus on the sustained activity of group I mGlu signaling and intracellular mechanisms underlying the persistent effect of receptor activation. We will review current knowledge regarding the significant role of the intracellular scaffold, Homer1a, in constitutive activity of group I mGlus. Further, we will discuss recent findings of *β*-arrestin function in sustained G protein activity in the intracellular GPCRs, addressing its possible relevance to the persistently active mGlu signaling. We conclude with a discussion of intracellular mGlu function and the suspected role of downstream mitogen-activated protein kinase (MAPK) signaling in the maintenance of sustained mGlu activity.

## 2. Persistent Action of the mGlus following Activation

The persistent activation is a common phenomenon among GPCRs [9, 20, 21]. Sustained G protein signaling after agonist washout has been reported for many GPCRs. This long-lasting action can be derived from the persistent effect of downstream cascades following agonist binding to the receptor, and/or agonist-independent persistent activation of the receptor itself. Previous studies have shown that the activation of mGlu downstream cascades exerts long-lasting influences on glutamatergic synaptic transmission, and persistent changes in synaptic efficacy elicited by mGlu activation are reversibly suppressed by mGlu antagonists [22]. For instance, a long-term depression (LTD) can be induced by the stimulation of group I mGlu using the agonist, 3,5-dihydroxyphenylglycine (DHPG), in hippocampal neurons. This group I mGlu-mediated LTD is fully or partially reversed by the application of the mGlu antagonists, such as *α*-methyl-4-carboxyphenylglycine (MCPG), 2-amino-2-(3-*cis* and *trans*-carboxycyclobutyl-3-(9-thioxanthyl)propionic acid (LY393053), *α*-amino-4-carboxy-2-methylbenzeneacetic acid (LY367385), or 2-methyl-6-(phenylethynyl)-pyridine hydrochloride (MPEP). The phenomenon is not specific to the group I mGlu. The group II and group III mGlu-mediated LTD is also reversed by representative antagonists [22]. These findings raise the possibility that prolonged alteration in neuronal activity induced by group I mGlu activation is mediated by the persistent activity of the mGlus themselves [14]. This suggested that the role of persistent activation can lead to modulation of neuronal activity in the physiological as well as pathological state [12, 14, 23].

The necessary condition for this persistent activity might differ based on the neuronal state and mGlu subtypes. In the case of the mGlu_5_ in CA3 hippocampal neurons, DHPG application at a sufficiently high temperature (30-31°C) for a sufficient period of time (>30 min) is necessary for the manifestation of persistent activation [15]. Under this condition, neuronal excitability was altered because of a change in state of the potassium channels and therefore persistent suppression of afterhyperpolarization (AHP), which is mediated by a p38 MAPK- and protein synthesis-dependent signaling pathway. The necessary condition (high temperature) in this case implicates that temperature-sensitive enzymes and/or ion channels might be involved in this mGlu_5_-mediated persistent AHP suppression [15, 24–26]. In the case of the mGlu_1_, the ion channel was transiently affected but the persistent change of state was not elicited by the same stimulation [15]. Interestingly, another study has reported that the persistent CA3 neuronal responses to the group I mGlu agonist DHPG were reversed by mGlu_1_ antagonist LY367385 or (hydroxyimino)cyclopropa[b]chromen-1a-carboxylate ethyl ester (CPCCOEt) and to a lesser extent by the mGlu_5_ antagonist MPEP, indicating that the mGlu_1_ is primarily involved [14]. In spite of the inconsistency, these studies commonly implicate the persistent activity and functional relevance of group I mGlu to long-lasting changes in neuronal activity.

## 3. Constitutive, Agonist-Independent Activity of mGlus

Many GPCRs exhibit agonist-independent activity. Although the exact mechanisms underlying the sustained signaling of GPCRs have not been fully understood, many investigations on the phenomenon have revealed that constitutive activity is an intrinsic feature of GPCRs [7–10]. The sustained activation of GPCRs can be modulated by signaling molecules, as well as endogenous ligands, and plays a significant role in maintaining both physiological and pathological state.

Group I mGlus have been reported to show constitutive activity [11, 12, 23, 27]. As a GPCR, the mGlu also has intracellular domains that can interact with numerous kinases, phosphatases, and proteins. These molecules modulate the action of the receptors, and many of them are shared by other GPCR signaling pathways. Constitutive activity of mGlus can result from changes in receptor conformation induced by these interacting molecules. Previous studies showed that mutation of specific allosteric binding domain residues results in conformational changes and modulates the constitutive activity of mGlus [28, 29]. Recently, it has been revealed that the constitutive activity of group I mGlus can be modulated by mGlus coupling to specific intracellular interacting molecules, such as the Homer family of scaffold proteins [11, 13].

## 4. Involvement of Homer Proteins in the mGlus Constitutive Activity

In the case of mGlu, the involvement of the Homer family of intracellular proteins is the most studied mechanism of the constitutive activity. Homer proteins are intracellular scaffolding proteins that interact with various membrane receptors including mGlus [30–32]. With the conserved Ena/VASP homology (EVH) 1 domain, Homer proteins bind to the C-terminal PPXXF motif of receptors and act as a scaffold for various intracellular effector interactions. The Homer family comprises of many alternative splicing variants from three Homer genes, and these multiple isoforms can be categorized into either long-form or short-form Homer proteins. The long-form Homer proteins (Homer 1b, 1c, 2, and 3) have a coiled-coil domain and form dimers with other intracellular effectors. The short-form Homer protein (Homer 1a), in contrast, only has an EVH1 domain and lacks a coiled-coil domain. Homer1a acts as a dominant-negative competitor for other long-form Homer proteins by binding to the receptors and disrupting intracellular signaling. The Homer1a is expressed in an activity-dependent manner, whereas other long-form Homer proteins are constitutively expressed. Homer1a is believed to counteract the hyperexcitability of neurons and thus play a key role in endogenous neuroprotection [32–36].

Other than such a homeostatic regulatory role, Homer1a is also involved in the constitutive activation of the mGlu [11, 13]. As a dominant-negative competitor for other long-form Homer proteins binding to the mGlu, Homer1a disrupts mGlu-Homer3 interaction when expressed. Because the Homer3 is constitutively expressed and acts as a negative regulator of mGlu constitutive activity via stabilization of the receptor, disruption of mGlu-Homer3 binding by Homer1a induction results in development of neuronal conditions for mGlu constitutive activation [11].

Although the involvement of Homer1a in the constitutive activity of mGlu has been reported, this concept does not clarify the basal mechanisms underlying constitutive activation of mGlu. The Homer1a mechanism for the induction of constitutive activity depends on its dominant-negative effect on mGlu-Homer3 binding. The study of Homer1a-mediated constitutive activity of mGlu was performed in the cerebellum, where basal Homer3 expression is known to be high [13]. As the expression of Homer3 differs depending on the brain regions and neuronal subtypes, it is speculated that the induction of constitutive mGlu activity by Homer1a might be inconsistent depending on the cellular condition. If Homer3 binding stabilizes mGlu and blocks the constitutive activation of the receptor, and Homer1a induces mGlu constitutive activity by disrupting mGlu-Homer3 binding, it is not appropriate to state that Homer1a is a necessary condition for the constitutive activity of mGlu. Thus, in the neuronal condition where Homer3 is absent, the mGlu constitutive activity might be conserved even without the presence of Homer1a. Rather, regarding the original Homer1a action of disrupting the binding of the mGlu to various interacting molecules, Homer1a would prevent the activation of certain intracellular pathways downstream in the mGlu signaling pathway. For instance, the disruption of mGlu interaction with Homer1b/c or Homer2 would affect calcium signaling and MAPK phosphorylations [37–39]. The degree of interruption in the mGlu downstream pathways by Homer proteins varies among different neurons and on the composition of the signaling pathways [39]. In that, the expression of Homer1a can decrease [40–42] as well as increase [13, 41, 43] the rise in calcium levels in response to mGlu stimulation, depending on the neuronal subtype [39]. Furthermore, the stimulation of mGlus activates several downstream pathways [16, 44], and Homer binding to mGlu does not uniformly activate or deactivate all these pathways [44]. Therefore, the functional effect of Homer on the sustained downstream activation of the mGlu might be pathway specific.

## 5. The Role of the *β*-Arrestin Pathway

We speculate that *β*-arrestin might be involved in the modulation of mGlu activity. In the classical view, *β*-arrestin had been regarded as a terminator of GPCR activity. According to this classical concept, agonist activation of the surface GPCR leads to GPCR kinase- (GRK-) induced phosphorylation of the receptor, followed by *β*-arrestin binding, and the binding of *β*-arrestin to the receptor results in desensitization and internalization of the receptor [21]. However, it is now clear that the action of *β*-arrestin is not limited to the desensitization or internalization of the receptor [45]. *β*-Arrestin acts as an adaptor or a scaffold, and its binding to the GPCR can activate signaling pathways independent of the G protein, to induce cellular change [46, 47]. *β*-Arrestin interacts with most of the GPCRs including mGlus [16, 17]. A recent study showed that the *β*-arrestin-induced G protein-independent signaling pathways of group I mGlu play a significant role in LTD in hippocampal neurons, and the involved pathways differ between CA1 neurons and CA3 neurons [17]. The authors of the study found that genetic ablation of *β*-arrestin2 results in deficits in LTD mediated by mGlu_1_ in CA3 neurons and by mGlu_5_ in CA1 neurons. They also have reported that the *β*-arrestin2 knockout mice have a deficiency in long-term potentiation (LTP) induced by low-frequency stimulation, paired stimulation of mossy fiber inputs to CA3 pyramidal neurons [48], but not in LTP induced by high-frequency stimulation [17]. An early study of CA3 pyramidal neurons revealed that NMDA receptor potentiation by mGlu_5_ is mediated by a G protein-dependent pathway, whereas potentiation by mGlu_1_ is mediated by a G protein-independent pathway [49]. The study demonstrated that the DHPG application could induce LTP under conditions of G protein blockade using GDP*β*S. This DHPG-LTP was blocked by the Src inhibitor. The authors discussed that the *β*-arrestin-mediated recruitment of Src kinase underlies the G protein-independent action of mGlu_1_ [49, 50]. Therefore, we can speculate that the *β*-arrestin downstream pathways of mGlu might be in an active state even under circumstances in which mGlu ceased its G protein-dependent pathways.

**Figure 1:**
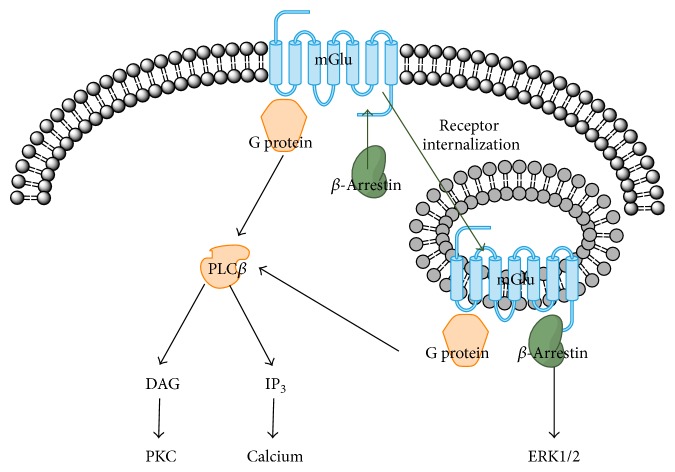
Proposed model of sustained mGlu activation. *β*-Arrestin binding to the mGlu internalizes the receptor. With stable binding to the mGlu C-terminal, *β*-arrestin mediates receptor internalization and activates ERK1/2 MAPK, but does not induce desensitization of G protein signaling. In this condition, the *β*-arrestin tightly binds only to the C-terminal tail but not to the transmembrane region, and thus the G protein can simultaneously bind to the transmembrane core of the receptor. The downstream pathways are activated without interfering with each other. The convergence of the downstream pathways on common effectors and their reciprocal interactions are omitted in this figure.

In addition to the activation of G protein-independent signaling pathways, the coupling of *β*-arrestin to mGlus might determine the activity status of the receptors. According to previous studies, GPCRs with weak coupling to *β*-arrestin (class A GPCRs) interact transiently with *β*-arrestin due to relatively low affinity and thus are recycled back to the plasma membrane shortly after endocytosis. GPCRs with stronger binding affinity to *β*-arrestin (class B GPCRs), on the other hand, show stable coupling and thus have been thought to experience endosomal degradation following *β*-arrestin-induced endocytosis [9, 20, 51]. Recent studies, however, have challenged this classical concept of *β*-arrestin-mediated cessation of GPCR activity. According to the studies, the binding of *β*-arrestin to GPCRs results in sustained activity of the G protein, mainly in the internalized GPCRs [8, 9]. In this new concept, *β*-arrestin and the G protein can bind simultaneously to the GPCR. This is achieved by *β*-arrestin binding to the C terminus and the G protein binding to the transmembrane core of the receptor [9]. The binding of *β*-arrestin to the C-terminal tail mediates receptor internalization and intracellular signaling, but does not induce desensitization of G protein signaling [8, 9, 20]. Thus, the high affinity of the C-terminal tail of the class B GPCRs to *β*-arrestin allows for the condition in which the G protein couples with the transmembrane core and simultaneously, *β*-arrestin couples with the C-terminal, which results in internalization of the receptor by *β*-arrestin and conserved G protein signaling in the internalized receptor [9, 20]. Consequently, the simultaneous activation of G protein-dependent and G protein-independent signaling pathways can occur in the internalized GPCR [9]. Although the interaction status of the transmembrane core and C-terminal tail to the G protein and *β*-arrestin in active mGlu is unclear, *β*-arrestin-mediated sustained signaling in the internalized GPCRs suggests a feasible mechanism for the constitutive activity (Figure 1).

*β*-Arrestin is also critically involved in modulating the plasticity of glutamatergic synaptic transmission [16, 17]. A recent study showed that the *β*-arrestin pathway is required for a certain type of group I mGlu-mediated plasticity, which involves the extracellular signal-regulated kinase (ERK) pathway and is mediated by mGlu_1_ in the CA1 neurons and mGlu_5_ in the CA3 neurons [17]. We speculate that *β*-arrestin is further involved in the constitutive activity of the mGlus.

## 6. Involvement of Intracellular mGlu_5_

Recently revealed intracellular activity of mGlu_5_ supports the idea above. According to the studies, more than 60% of mGlu_5_ are located in the intracellular site [52, 53], and activation of the intracellular mGlu_5_ leads to sustained cytosolic calcium responses [53–56]. Regarding the *β*-arrestin-mediated sustained activity of GPCR that takes place with receptor internalization, the high composition ratio of intracellular mGlu_5_ inspires the idea that the intracellular mGlu_5_ activity is correlated with *β*-arrestin binding and sustained receptor signaling.

This intracellular mGlu_5_ activity plays a significant role in maintaining physiological and pathological plasticity during hippocampal LTD [54] and nerve injury-induced hyperexcitability of spinal neurons [53]. Interestingly, the signaling cascades induced by intracellular mGlu_5_ activation are distinct from the downstream signaling of mGlu_5_ in the plasma surface membrane [55, 56]. Only intracellular mGlu_5_, not surface membrane mGlu_5_, causes ERK1/2 phosphorylation. This was demonstrated by the upregulation of ERK1/2 phosphorylation in response to the treatment of membrane-permeable agonist, quisqualate, in the presence of impermeable, nontransported antagonist, LY393053. The quisqualate-mediated upregulation of ERK1/2 phosphorylation could be blocked by the membrane-permeable antagonist MPEP. Conversely, the impermeable, nontransported agonist, DHPG, could not induce an increase in ERK1/2 phosphorylation. Similar discrepancies regarding ERK1/2 activation have been shown in a recent study of the *β*-arrestin-dependent downstream signaling pathway of mGlu_5_ activation [16].

## 7. ERK1/2 MAPK Pathway

In the signaling cascades of many GPCRs, G protein and *β*-arrestin-mediated pathways share common downstream effectors of ERK1/2 MAPK [57–59]. The binding of *β*-arrestin to activated GPCRs contributes to ERK1/2 phosphorylation, and sustained phosphorylation of ERK1/2 promotes GPCR internalization and constitutive signaling [57, 59–63]. In the case of the mGlu_1/5_, agonist stimulation of the receptor results in ERK1/2 phosphorylation, which plays a significant role in the synapse [64–66]. This ERK1/2 activation is unaffected or only partially affected by inhibitors of PLC [38], which is a downstream effector of the G protein-mediated pathway [46]. Recent studies showed that mGlu_5_-mediated ERK1/2 activation was completely blocked by genetic reduction of *β*-arrestin2 [16, 17]. This suggests that the mGlu_5_-mediated ERK1/2 activation is *β*-arrestin pathway-dependent but not G protein pathway-dependent [16]. As discussed above, this biased involvement for the phosphorylation of ERK1/2 is a shared characteristic in studies of intracellular mGlu_5_ activation and mGlu_5_-mediated *β*-arrestin signaling pathway.

**Figure 2:**
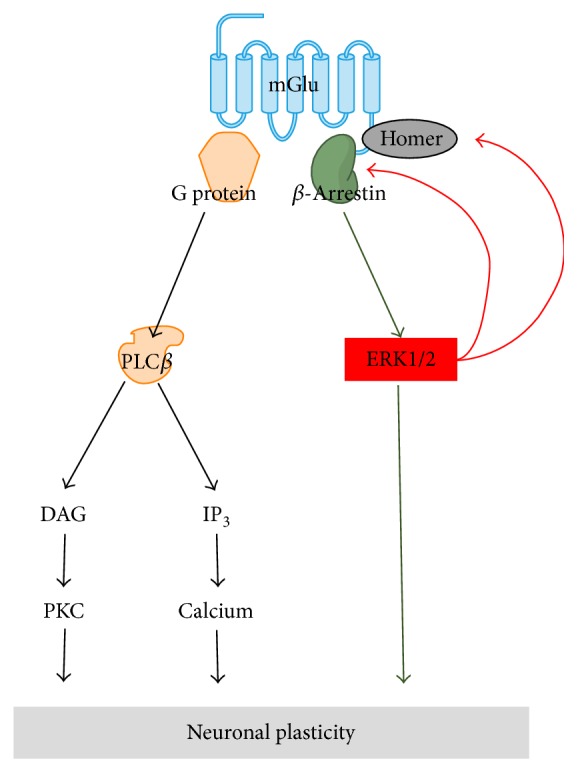
Feedback modulation of mGlu activity by ERK1/2. Stable binding of *β*-arrestin to the receptor upregulates ERK1/2 activation, whereas transient binding of *β*-arrestin downregulates it. The activated ERK1/2 preferentially phosphorylates receptor-bound *β*-arrestin and regulates its function. In addition, the activated ERK1/2 phosphorylates the Homer binding site of mGlus C-terminal. These feedback mechanisms might play a role in keeping the mGlu persistently active.

Interestingly, activated ERK, in turn, regulates the binding of *β*-arrestin and Homer proteins to the receptor. The actions of *β*-arrestin on the GPCRs are regulated by an ERK-mediated feedback mechanism, as activated ERK preferentially phosphorylates receptor-bound *β*-arrestin [46, 67, 68] and regulates its function [62]. Furthermore, activated ERK1/2 phosphorylates the serine-proline motif of mGlu_1_ and mGlu_5_, and the phosphorylation sites include the Homer binding site of mGlus C-terminal [44, 69]. Thus, it is likely that once the *β*-arrestin pathway of intracellular mGlu is sufficiently activated, subsequent ERK activation would affect receptor coupling to *β*-arrestin and Homer proteins and eventually modulate the downstream signaling of mGlus (Figure 2). Whether the ERK-induced phosphorylation of Homer binding site of mGlu results in activation or deactivation of mGlu signaling might be case specific, as Homer modulation of mGlu signaling would differ based on neuronal conditions [39, 69]. We propose that, under certain circumstances, binding of Homer and *β*-arrestin to the receptor adjusted by kinase phosphorylation would lead to sustained activation of the mGlu.

## 8. Regulation of the Interactions

The coupling of the Homer proteins to the receptor is affected by phosphorylation of the binding sites. In group I mGlu, the proline-directed kinases, such as ERK1/2 and cyclin-dependent kinase (CDK) 5, phosphorylate group I mGlu at the Homer binding site and control the downstream signaling pathways [44, 70]. A multidomain scaffolding protein called Preso1 binds these proline-directed kinases and regulates Homer-mGlu binding [44]. Furthermore, expression of Homer1a after the induction of LTP in neurons present in the hippocampal dentate gyrus requires the ERK1/2 cascade [71]. As such, the interaction between kinases and proteins plays an important role in regulating the expression of Homer proteins and their interaction with mGlus. Since the Preso1-mediated regulation of Homer binding does not influence the surface expression of mGlus [44], it is unlikely that the downstream activation of the Homer-mediated mGlu directly recruits *β*-arrestin. Rather, it is speculated that the Homer-mediated and the *β*-arrestin-mediated pathways affect each other by the phosphorylation of the receptor and each protein. Importantly, the proline-directed kinases, which mediate mGlu phosphorylation at the Homer binding site, can be activated by numerous signaling pathways and are not specific to the receptor. This suggests the possibility of receptor crosstalk [44] and interaction with *β*-arrestin signaling. The binding of *β*-arrestin to the mGlus critically affects ERK1/2 activation via Raf signaling and protein synthesis following receptor activation. In return, *β*-arrestin signaling is affected by the ERK-mediated feedback control [62]. Interestingly, *β*-arrestin has two different modes of action in the regulation of ERK. A recent study of M_1_ muscarinic acetylcholine receptors revealed the bidirectional control of ERK by *β*-arrestin binding to the receptor, showing that the stable binding of the *β*-arrestin upregulates ERK1/2 expression, whereas transient binding downregulates it [72]. Although details regarding *β*-arrestin binding to the mGlus during receptor activation are still unclear, this raises the prospect that the sustained activity of the mGlu is regulated by the functional interaction between Homer proteins and *β*-arrestin, which is balanced by ERK activation.

## 9. Effects of the Interactions

Proteins responsible for long-term expression of synaptic plasticity are rapidly translated in response to mGlu activation. Disrupted regulation, as well as excessive protein synthesis, can result in neuronal disorders [73]. Regarding the role that activated ERK1/2 plays in the regulation of gene expression, the signaling cascades involved in ERK activation would directly affect mGlu-mediated protein synthesis.

Although the binding of Homer1a to the group I mGlu leads to constitutive activation in certain circumstances [13], the consequence is manifested by the G protein-dependent downstream cascades, and not the ERK activation cascade. We speculate that the constitutive downstream activation of the G protein cascades is just one of the many possible consequences of Homer1a binding to mGlu. This view is supported by the effects of Homer proteins and *β*-arrestin on the Ras-mediated activation of the ERK1/2. The Ras protein transduces signals from activated GPCRs to the cytoplasm and nucleus and contributes to the induction of various effector molecules, including MAPKs [74]. In many GPCRs, the Ras-dependent activation of the ERK1/2 MAPK pathway requires Src kinase signaling [59], and the interaction between *β*-arrestin and Src kinase plays an important role in this GPCR-Src-ERK1/2 pathway [49, 70]. In addition, *β*-arrestin directly binds to c-Raf [68] and relieves the autoinhibition of the kinase even without Ras, which leads to the activation of the ERK cascade [75]. In the case of the mGlu, it has been proposed that *β*-arrestin acts as a scaffold to couple the Src kinase to the activated mGlu [17, 49, 71] and thus is required for mGlu-mediated ERK1/2 activation [16]. Interestingly, upon activation of the mGlu_5_ in striatal neurons, only a small portion of ERK1/2 is activated by the PLC*β*/IP_3_/calcium-dependent pathway [38], which is a G protein-mediated cascade [16]. In the same condition, much stronger ERK1/2 activation is achieved by the calcium-independent pathway, in a Homer1b/c-dependent manner [38]. Since the ERK1/2 activation is *β*-arrestin pathway-dependent, this implicates the crosstalk between the Homer1b/c and *β*-arrestin downstream pathways. In this neuronal condition, Homer1a binding to mGlu would negatively regulate ERK1/2 activation via inhibition of Homer1b/c binding to mGlu. Indeed, Homer1a strongly attenuates mGlu-dependent activation of ERK1/2 in the spinal cord [40]. Notably, the disruption of mGlu-Homer interactions selectively blocks the phosphoinositide 3-kinase- (PI3K-) Akt-mammalian target of rapamycin (mTOR) pathway but not the ERK pathway in hippocampal neurons, suggesting the region-specific role of Homer in mGlu signaling [76]. As a read-out of the protein synthesis downstream of mGlu_5_ activation, the ERK change implicates distinct mode of actions of the mGlu_5_ following its interaction with Homer proteins.

The functional consequences of the interaction are manifested by various physiological and pathological responses in neurons. The glutamate-induced protective signaling of mGlu_1_ is mediated by sustained, *β*-arrestin-mediated ERK activation [77]. In the case of Homer, the binding of the Homer protein to the phosphoinositide 3 kinase enhancer (PIKE) following quisqualate- or DHPG-induced activation of group I mGlu activates PI3K and prevents neuronal apoptosis [78]. Therefore, disruption of this interaction would affect the basal viability of the neuron. In addition, the mechanisms of the group I mGlu-mediated synaptic plasticity involve *β*-arrestin [16, 17] and the Homer protein [76]. These interactions are associated with neuronal diseases such as fragile X [16, 27, 76], chronic pain [40, 79, 80], and addiction [81, 82]. Although these findings suggest the involvement of sustained activation, direct implication of sustained mGlus activity in the regulation of the synaptic transmission has not yet been established.

## 10. Conclusion

Constitutive activity of mGlu plays a critical role in neuronal responses. The coupling of mGlus to effector molecules including G proteins or *β*-arrestins not only mediates downstream effectors but also determines the activity of the mGlus itself. These effectors coupling to the mGlus and activation following downstream pathways could be modulated by reciprocal interactions between the binding molecules including kinases, phosphatases, and proteins. We propose that the Homer proteins, ERK1/2 MAPK, and *β*-arrestin affect each other and regulate the constitutive activity of mGlu. This regulation would occur in the internalized mGlu following sufficient receptor activation, and the C-terminal binding to interacting molecules would modulate the implementation of downstream signaling.
